# A Stacked Neural Network Model for Damage Localization

**DOI:** 10.3390/s24217019

**Published:** 2024-10-31

**Authors:** Catalin V. Rusu, Gilbert-Rainer Gillich, Cristian Tufisi, Nicoleta Gillich, Thu Hang Bui, Cosmina Ionut

**Affiliations:** 1 Department of Computer Science, Babeș-Bolyai University, Str. M. Kogălniceanu 1, 400084 Cluj-Napoca, Romania; thuhang.bui@ubbcluj.ro (T.H.B.); cosmina.ionut@stud.ubbcluj.ro (C.I.); 2 Department of Engineering Science, Babeș-Bolyai University, Str. M. Kogălniceanu 1, 400084 Cluj-Napoca, Romania; gilbert.gillich@ubbcluj.ro (G.-R.G.); cristian.tufisi@ubbcluj.ro (C.T.); nicoleta.gillich@ubbcluj.ro (N.G.); 3 Doctoral School of Engineering, Babeș-Bolyai University, Str. M. Kogălniceanu 1, 400084 Cluj-Napoca, Romania

**Keywords:** damage detection, stacking techniques, ANN, natural frequency, ANN, LSTM, MLP, model comparison

## Abstract

Traditional vibration-based damage detection methods often involve human intervention in decision-making, therefore being time-consuming and error-prone. In this study, we propose using Artificial Neural Networks (ANNs) to detect patterns in the structural response and create accurate predictions. The features extracted from the response signal are the Relative Frequency Shifts (RFSs) of the first eight weak-axis bending vibration modes, and the predictions refer to the damage location. To increase the accuracy of the predictions, we propose a novel stacked neural network approach, capable of detecting damage locations with high accuracy. The dataset used for training involves, as input data, the RFSs calculated with an original method for numerous damage locations and severities. The following models were used as building blocks for our stacked approach: Multilayer Perceptron, Recurrent Neural Network, Long Short-term Memory, and Gated Recurrent Units. The entire beam was thus split into segments and each network was trained in this stacked model on one beam segment. All results obtained with the models are also compared to a standard neural network trained on the entire beam. The results obtained show that the model that performs the best contains 14 stacked two-layer feedforward networks.

## 1. Introduction

Actual progress allows for fast and efficient damage detection using non-destructive local techniques such as ultrasound testing, thermography, acoustic emissions, and vibration-based methods [1]. Another class of non-destructive techniques is based on the analysis of vibrations. However, few such techniques can accurately predict the damage locations and extent, mainly because of the complexity of data acquired during the monitoring process. A viable solution to overcome this difficulty is using Artificial Neural Networks (ANNs), which can handle inverse problems with high accuracy and robustness [2,3]. Solving an inverse problem requires the models to be able to extract information about the physical object, in this example, a beam, using just the measurements obtained from laboratory experiments and simulations. These neural networks can identify patterns from the information they receive and learn in time to make accurate predictions. Identifying the optimal features to be used as input data for the models is vital to enable them to learn and predict damage positions as accurately as possible. This study aims to determine an optimal network model and topology to localize cracks accurately. To achieve this, we trained different types of neural networks and test their performance on a set of data to determine which of the networks has the best accuracy. Additionally, we used performance improvement techniques such as model stacking and hyperparameter tuning to create very accurate predictions. Utilizing a stacking method, we improved prediction accuracy by dividing the training data into subdomains specific to the different beam segments.

Vibration-based damage detection (VBDD) has become an important research topic because it allows monitoring the integrity of a structure during operation. These methods associate the damage parameters (location, extent/depth, and type) with the changes in the response signal. This signal carries information about the modal parameters of the structure, among which the most relevant are the natural frequencies and the mode shapes. The damages evaluated with VBDD techniques include cracks, delamination, and corrosion [4]. 

Because of the advantages of VBDD techniques, these widespread and numerous studies have been conducted to determine the best approach to assess the damage location accurately when small changes in the response signal are present [1]. We found that numerous techniques associate non-destructive methods with Artificial Neural Networks. 

Senthikumar et al. [4] proposed using a feedforward multilayer back propagation neural network to detect surface cracks, edge cracks, and delamination. They researched composite beams, which have applications in many industries. Using vibrations and measuring the changes in natural frequency, they determined if damages were present. The Artificial Neural Network predicted the crack location length based on natural frequency measurements. The ANN had three layers, took the first three natural frequency change rates as input, and gave the crack location and the length as output. The hidden layer consisted of 12 neurons. There were 104 samples; 70% were used for training, 15% for validation, and the other 15% for testing the network. The crack length was predicted with a maximum deviation error of 5,9%, whereas the location prediction succeeded better, with a maximum error of 2.16%. 

Sreekanth et al. [5] proposed a similar approach to determine the delamination location and size of the delaminated layer. They used a feedforward multilayer backpropagation Artificial Neural Network with three layers. The ANN took five natural frequencies as input data, had one hidden layer with eight neurons, and had three outputs (location, layer, and delamination size). The damaged layer was correctly identified for all four test cases. At the same time, the location was predicted with a maximum deviation error of 1.46%, and the delamination size had a maximum error of 1.1%.

Another relevant study is presented by Saeed et al. [6]; it focuses on crack identification in curvilinear beams using ANNs and an adaptive neuro-fuzzy inference system (ANFIS). Natural frequencies and frequency response functions were computed, and changes in the frequencies of the breams signify damages and can be used to determine the crack size and location. The prediction error is for multiple ANN models smaller than for a single ANN or multiple ANFIS. At the same time, they showed that cracks more minor than 5 mm can be harder to be detected, while the accuracy in predicting the location decreases. The accuracy also decreases with the increase in the noise level, but the ANFIS models are less sensitive to noise than the ANN models.

Tran-Ngoc et al. [7] show that the cuckoo search (CS) algorithm can help to improve the training parameters when using Artificial Neural Networks for damage detection. One major drawback of backpropagation and other gradient descent-based algorithms is that learning can quickly converge to a local minimum [8]. To address this, the authors propose an evolutionary algorithm based on global search coupled with an ANN to help improve convergence and bias. The study focuses on defect identification in bridges and beam-like structures utilizing various evolutionary algorithms, such as cuckoo search, particle swarm optimization, and genetic algorithms. After benchmarking these algorithms on different datasets, they concluded that the one that performed the best was cuckoo search with Artificial Neural Networks because it can accurately identify the damage location and severity. Evolutionary algorithms can find alone the location and severity accurately, but on large datasets, they take a long time to converge. Therefore, using ANN-CS is the best combination because it localizes and quantifies the damage accurately and has the best convergence speed.

Shilei Zhang et al. [9] analyzed and compared some algorithms to determine which would best suit damage detection. They considered that the natural frequency is easy to obtain and has a good measuring accuracy, but more is needed to determine the location and damage degree. On the other hand, using mode shapes as input parameters would provide sufficient information, but there would be a significant measuring error. The structure of the Artificial Neural Network and the algorithm used significantly impact the accuracy of the damage detection. In this study, the authors compared an adaptive variable step size algorithm with the Levenberg–Marquardt and a Homogeneous algorithm. The Levenberg–Marquardt algorithm is the best solution out of the abovementioned algorithms, and together with the modal parameters, it could accurately identify damage.

Damage detection in beam-like structures supported by machine learning is also analyzed by Aydin and Kisi [10]. They compared Multilayer Perceptron (MLP) with radial bias neural networks (RBNNs) to determine the best-suited approach for defect detection. Firstly, based on beam properties, the first four frequencies are predicted using the neural networks, and then for the second part of the study, the crack location and depth are predicted based on frequencies, modes, shapes, and beam properties. They tested many values for hidden neurons and epochs to find the best configuration. Based on this analysis, the MLP with 100 hidden nodes and 1000 epochs and the RBNN with 2000 hidden nodes and 0.7 spread performed the best. The RBNN model also predicts the crack location and depth better than the MLP model since the RBNN has a better interpolating capability in multidimensional space. However, when tested in the presence of noise, the relative error for the MLP is less than that of the RBNN model.

The shortcoming of the actual vibration-based damage detection methods that involve ML is the lack of reliable mathematical models for cracked beams that can predict the frequency changes due to damage. Without such models, the input data for training the neural network are difficult to obtain or implies extensive work. Another aspect that was not considered in actual studies is the division of the beam into segments and the training of the networks for the specific segments.

Our previous research [11,12,13,14] used vibration-based techniques to identify damage characteristics such as location and depth. The developed damage detection methods are based on original mathematical relations that make a link between the damage parameters and the frequency changes that occurred due to the damage [15]. Due to complexity, identifying patterns can be challenging. Therefore, we now involve the deduced mathematical relations and different types of neural networks to identify damage. As a novelty, we train the networks for stacked beam segments to improve the prediction accuracy. 

Combined models such as stacked Multilayer Perceptron, Recurrent Neural Network, Long Short-term Memory, and Gated Recurrent Units are proposed, and the experimental results verified the effectiveness of the method. Multilayer Perceptrons (MLPs) are essential to modern damage detection techniques as they provide a baseline model that facilitates direct comparisons with other state-of-the-art approaches in the field. They can be especially useful for detecting damage-related features in structural data obtained from Finite Element Method (FEM) simulations, where temporal dependencies are not a major concern, due to their capability to process static or non-sequential data efficiently and their strong feature extraction skills.

The ability of the model to identify complex patterns and relationships in the FEM data is improved with the addition of RNNs, LSTMs, and Gated Recurrent Units (GRUs). While temporal dependencies are not the main focus of this study, these designs are excellent in modelling non-linear interactions between different damage indicators, which may reveal relationships that simple feedforward models like MLP might overlook. Both LSTM and GRU, which were designed expressly to overcome drawbacks like the vanishing gradient issue that normal RNNs face, were assessed for their capacity to process data, where subtle variations may indicate potential damage. This capability is critical as models can assist in detecting subtle structural anomalies that typical MLP models may disregard.

This paper is organized as follows. In Section 1, we present the state of the art in detecting damage using vibration-based methods coupled with machine-learning techniques. In Section 2, we describe the methods used for analytically generating training data and the employed methods for generating test scenarios by using FEM and experimental analysis. Section 3 presents the development of the ML models, including Basic and Stacked neural models. The obtained results are presented in Section 4, where a thorough comparison is made between the basic and stacked ML methods by considering analytic, FEM, and experimental cases. The conclusion section summarizes the study’s findings on identifying damaged locations using various neural network architectures. This research demonstrates that deep learning can accurately predict damage positions, achieving high accuracy in most cases.

## 2. Materials and Methods

### 2.1. Types of Neural Networks Involved in This Study

This section provides an overview of the dataset used in this study and the methodology used to implement the models and their enhancements. Furthermore, different network architectures are compared, including Multilayer Perceptron and Long Short-term Memory networks. Additionally, we explore different approaches, such as dataset segmentation to train multiple models on distinct segments, model stacking, and parameter optimization to improve the model performance [16].

Artificial Neural Networks (ANNs) model both linear and non-linear relationships and improve through training. A typical ANN has an input layer, hidden layers where weights determine input–output relationships, and an output layer. The most common type is the Multilayer Perceptron (MLP) with backpropagation for supervised learning. Overfitting is a key issue, often addressed by early stopping [17]. 

Recurrent Neural Networks (RNNs) handle sequential data and model dependencies by passing information through loops [18]. They use backpropagation through time (BPTT) but suffer from exploding gradients [19]. LSTMs improve RNN performance with input, forget, and output gates, enhancing long-term memory [20]. BiLSTMs and GRUs are additional variants, with GRUs offering faster computation but slightly lower performance than LSTMs [21].

Choosing the best architecture depends on the problem and dataset, with LSTMs often favored for their balance of accuracy and efficiency. In the current study, Python is employed to develop machine learning models, including existing libraries such as TensorFlow, NumPy, and Matplotlib.

### 2.2. Generation of the Training and Testing Data 

To create a dataset for training the neural network models, we analytically generate data involving original mathematical relations [12] that permit calculating the Relative Frequency Shift (RFS) values for the bending vibration modes. Previous studies demonstrated that six to ten weak-axis bending vibration modes are necessary for conclusive and reliable predictions. In this study, we involve eight vibration modes. The procedure to calculate the RFSs is described below.

First, we calculate the natural frequencies of the beam with a crack using the equation:

(1) $$f_{i-D}\left(x,a\right)=f_{i-U}\{1-\gamma (a){\left[{\bar{\phi }}_{i}^{\prime \prime }(x)\right]}^{2}\}$$

In equation (1), the term *γ*(*a*) represents the severity of a transverse crack with depth *a*, and ${\bar{\phi }}_{i}^{\prime \prime }(x)$ is the normalized modal curvature at location *x* for the vibration mode *i*. Here, we calculate the frequency of the damaged beam relative to the frequency of the intact beam *f_i-U_*. 

The crack severity is determined using the method described in [13]; the mathematical relation to calculate *γ*(*a*) is as follows:

(2) $$\gamma \left(a\right)=\frac{\sqrt{\delta_{D}(0,a)}-\sqrt{\delta_{U}}}{\sqrt{\delta_{D}(0,a)}}$$

In Equation (2), we introduce the terms $\delta_{D}(0,\;a)$ and $\delta_{U}$, which represent the deflection of the beam with a crack at the fixed end and that of the intact beam.

At location *x*, the modal curvature of the cantilever beam, which we consider in this study, is calculated using the following relationship:

(3) $$\phi^{\prime \prime }\left(x\right)=\cosh\left(\lambda \frac{x}{L}\right)+\cos\left(\lambda \frac{x}{L}\right)-\frac{\cos\lambda +\cosh\lambda }{\sin\lambda +\sinh\lambda }\cdot \left[\sinh\left(\lambda \frac{x}{L}\right)+\sin\left(\lambda \frac{x}{L}\right)\right]$$

Now, from Equation (1), we calculate the RFSs for various damage scenarios involving the following relationship [14,15]:

(4) $$\Delta {\bar{f}}_{i}\left(x,a\right)=\frac{f_{i-U}-f_{i-D}(x,a)}{f_{i-U}}=\gamma (0,a)\cdot {\left[{\bar{\phi }}_{i}^{\prime \prime }(x)\right]}^{2}$$

We generate input data consisting of RFS values calculated using the equation (4) for the first eight vibration modes and many damage scenarios. These scenarios involve transverse cracks positioned at various locations along the beam and having different depths. The crack locations are evenly distributed along the beam, the distance between two neighbor cracks being *s* = 2 mm. Note that one crack affects the beam at a time. The positions remain the same when we train networks on specific segments. The dataset is structured to encompass transverse crack depths ranging from *a* = 0.5 mm to a maximum of *a* = 3 mm. The severities of the cracks are determined per the methodology outlined in [13]. The output data consist of the crack locations. The complete data used for training are available in the database [22].

Table 1 displays a training dataset excerpt, with eight modes representing the eight RFSs as input features. These RFSs are utilized for training all the proposed neural networks. A distinct model is trained for each segment to achieve precise location predictions; these locations are the output data.

### 2.3. Test Data Gathering

Two widely employed techniques, namely the Finite Element Method (FEM) and an experimental approach, are utilized to generate data to test the proposed procedure. These methods complement each other and provide distinct benefits in capturing the intricate dynamics of real-world systems.

#### 2.3.1. Test Data Generation Using the Finite Element Method (FEM)

**Figure 1:**
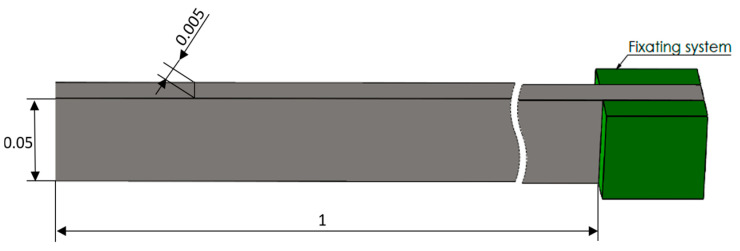
Main dimensions of the steel cantilever beam.

For generating various damage scenarios and to depict the natural frequencies of a steel cantilever beam in both undamaged and damaged conditions, FEM simulations are conducted in the ANSYS software program [15]. The ANSYS modeling capabilities are employed to define the geometric parameters of the beam, length of *L* = 1 mm, width of *B* = 50 mm, and height *H* = 5 mm; see Figure 1. The material assigned to the beam, structural steel, is characterized by a Young’s modulus of *E* = 2.1 × 10^5^ MPa and a density of *ρ* = 7850 kg/m^3^. We generate the mesh with hexahedral elements of a maximum edge size of 2 mm. Boundary conditions are applied to the right beam end to simulate the fixing conditions of the cantilever beam. 

The natural frequencies and the corresponding mode shapes of the intact cantilever beam are obtained using modal analysis. Once the natural frequencies of the undamaged beam have been calculated, the next step involves introducing an open transverse crack at various locations and depths along the beam to simulate the damaged state. Remeshing is carried out to accurately represent the damaged region and effectively capture the crack’s effects. Subsequently, another modal analysis is conducted to obtain the natural frequencies and mode shapes for different damage scenarios. 

The first eight transverse vibration modes are calculated using ANSYS while accounting for the various locations and depths of the open transverse crack. The resulting natural frequencies are recorded, and the corresponding RFS values are calculated using the frequencies of the intact and damaged beams. These RFS values are input values to test the neural network models. 

#### 2.3.2. Experimental Measurements

**Figure 2:**
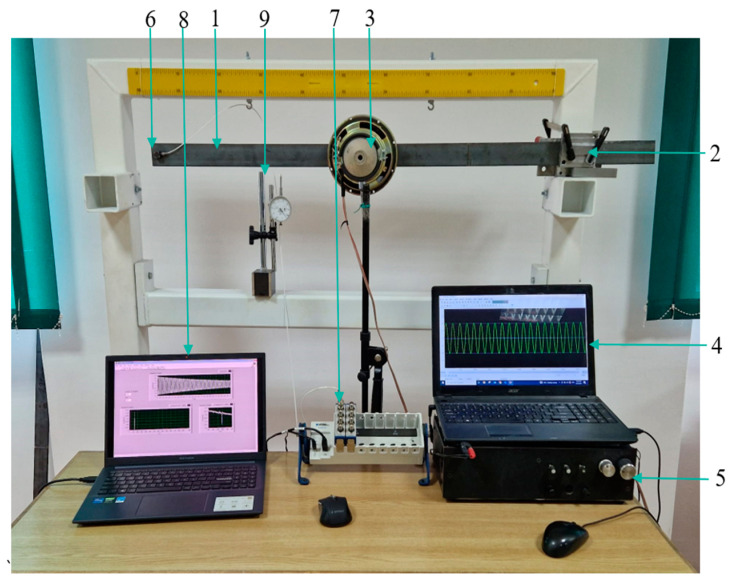
Experimental setup.

Supplementary test data are obtained from measurements. The experimental procedure is described in [15], and a general view of the setup is given in Figure 2. We measure the natural frequencies of the cantilever beam made of steel, which has the same dimensions as that used for numerical simulation. The cantilever beam (1) was rigidly fixed on the stand to ensure stability during testing. The rigid mounting (2) ensures a beam response that is very close to that obtained theoretically for the clamped-free condition. 

For the undamaged state, the cantilever beam underwent excitation by utilizing a loudspeaker (3) positioned at an antinode of the vibration mode for which we estimate the frequency. An audio software installed on the laptop (4) generates a signal with the desired frequency, which is transmitted through an amplifier (5) to the loudspeaker, causing vibrations in the beam. An accelerometer (6) is attached to the free end of the cantilever beam to capture the vibrational response. This accelerometer is connected to an NI 9034 chassis containing a data acquisition module (7), which interfaced with LabVIEW installed on the laptop (8). To accurately estimate the natural frequency, we use a Python software package [15] and a previously developed excitation technique [5]. Before starting the measurement procedure, to ensure a correct horizontal mounting of the beam in the clamping system, the height gauge system (9) is used.

To examine the impact of damage on the natural frequencies, we machined transverse cuts at predetermined locations on the cantilever beam. The cuts were made with great care to ensure consistency and reproducibility. Subsequently, the damaged beam was mounted using the fixing system, and the same excitation and data acquisition procedures were applied. With the natural frequencies of the beam in both states, we calculated the RFS values and utilized these to evaluate the accuracy of the developed intelligent models in detecting and locating damages based on real-world measurements. 

## 3. Stacked Approach

To tackle damage location detection with high accuracy, we propose a novel stacked approach composed of individual neural networks trained on a section of the beam in this study. We employed as base models: feedforward ANN, Recurrent Neural Network (RNN), Long Short-term Memory (LSTM), and Gated Recurrent Units (GRU). We also developed a basic feedforward neural network trained on the entire beam as a benchmark tool. 

### 3.1. Basic Neural Model

In line with other studies [4,5,10,23], we initially developed a feedforward multilayer backpropagation network trained on the entire beam length to predict the damage location. We started with a basic neural network structure, which has eight input nodes and two hidden layers, the first consisting of 100 nodes and the second one of 20 nodes and one output, representing the location of the damage. *ReLU* was used for the hidden layers and the *sigmoid* activation function for the output layer. The data were split into 80% for training and 20% for validation. A different set of data, consisting of 315 generated samples, were used for testing. All training and testing data are available at [22] and [24]. These samples consider damages generated with different depths and having the locations evenly distributed along the beam, with a distance of *S* = 5 mm between two consecutive damages. This ensures that the models are also tested on novel data. RMSProp was used as the optimization algorithm, and performance metrics such as mean squared error (MSE) and validation MSE were analyzed. The model was trained for 50 epochs and reached a validation mean squared error of 0.0019. After training the network with different parameter values and analyzing the results, significant errors are observed. Table 2 shows the first five samples with the highest error percentages, with the worst being 25.74%, which is a significant error when predicting the damage location. Additionally, for more than 50 samples, the error was greater than 3%. As expected, locations found at the ends of the beams were difficult to predict, but the model also had difficulties in predicting other positions, such as 0.333 and 0.837. 

### 3.2. Stacked Models

**Figure 3:**
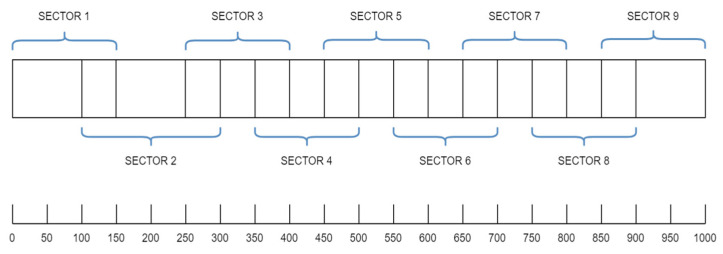
Configuration with 9 beam segments for which we trained NNs.

**Figure 4:**
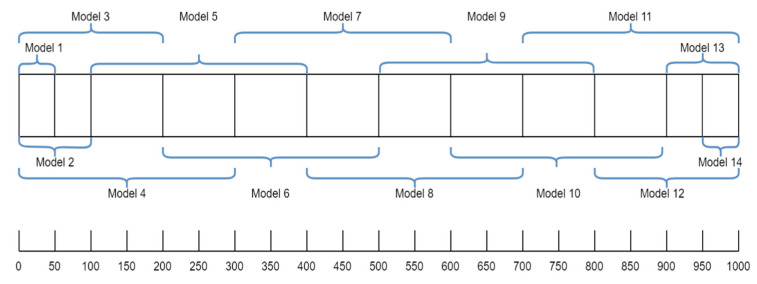
Configuration with 14 beam segments.

To address the shortcomings of the basic neural model, we developed and trained several stacked ANN models, each containing networks specialized on a specific subset of the beam. This means that each individual network is trained using the eight RFSs for its corresponding beam sector, and in the next step, each gives a prediction for the damage location, as shown in Figure 3. A regressor is then used to aggregate the individual predictions from each base model and output the final prediction, corresponding to the actual damage position. Three different configurations for the number of individual networks in a stacked model are considered: 9, 10, and 14 networks. The difference between them lies essentially in the degree of overlap. More overlap means that more than one model will be trained on a particular beam part. Figure 3 shows the beam sectors corresponding to 9 models, while Figure 4 shows the sectors corresponding to 14 models. 

We have found that overlapping led to bespoke models for smaller parts of the beam, which in turn led to an overall performance improvement. Additionally, each stacked model was implemented using ANN, RNN, LSTM, bidirectional LSTM, and GRU leading thus to 12 compared stacked models.

**Figure 5:**
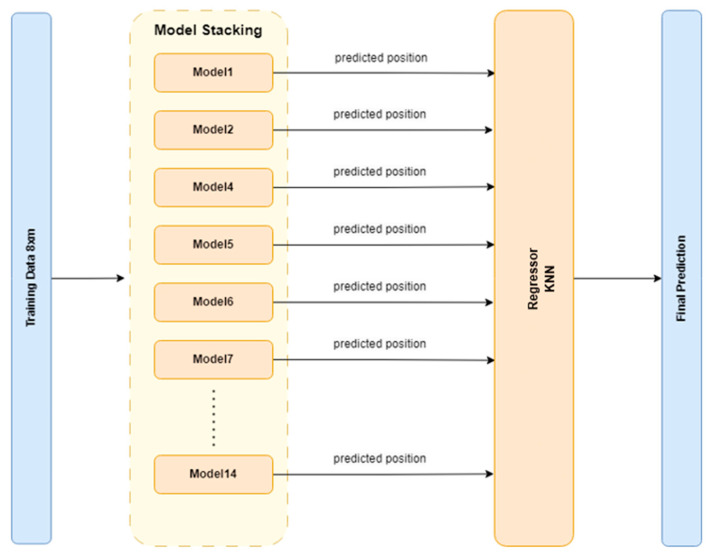
Stacked neural network model structure.

For the final step (Figure 5), several regressors were initially tested: KNN-Regressor, Linear Regressor, Decision Tree Regressor, and Support Vector Regression. The K-Nearest Neighbors Regressor was found to perform the best.

#### Hyperparameter Selection

While increasing the number of hidden layers for feedforward neural networks does not typically lead to better results, network performance is often dependent on a wide range of hyperparameter choices about the topology of the network, regularization, and optimization [25]. This is especially important because, for example, even the number of neurons in a network impacts performance. Too few neurons used in a network means that the network will not be able to solve the complex problem and have accurate predictions; on the other hand, too many neurons can lead to overfitting issues [26]. The hyperparameter selection analysis was performed on the stacked models using ANN and RNN [27]. 

**Figure 6:**
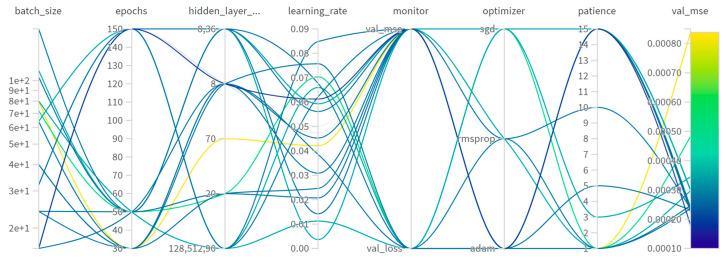
Comparison of different hyperparameter values for stacked MLPs.

To find the best combination of hyperparameter values, 20 different networks were created with different parameter values. These parameters and their values can be seen in Figure 6. In this case, the performance was given by the value of mean squared error (MSE) for the validation [28]. A Bayesian process was employed to speed up the search. 

Based on this analysis, the network which performed the best consisted of one hidden layer with 8 neurons and a second hidden layer consisting of 36 neurons. Out of the three optimizers, Adam performed the best, with the value for the learning rate being 0.0427. When training the model, a learning rate reduction algorithm was also used, having the monitor value *val_mse*. L2 regularization with a factor of a factor of 0.0001 was used to avoid overfitting. The network was trained for 300 epochs with a batch size of 100, and the value for the validation mean square error after 300 epochs was 0.0007691. Here we can observe a significant improvement from the basic network, which had a mean square error of 0.0019 [28]. Additionally, it can also be observed that the validation errors are higher for more than 2 hidden layers, meaning that having a more complex network architecture would not improve the prediction accuracy.

**Figure 7:**
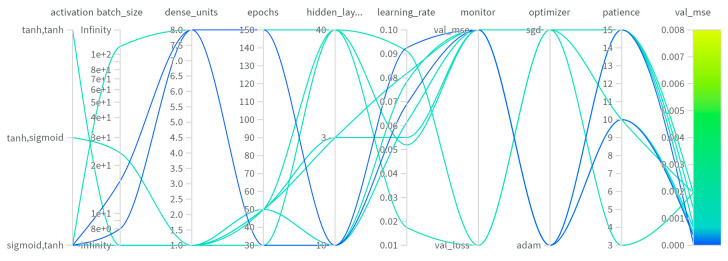
Comparison of different hyperparameter values for RNNs.

Different network hyperparameter configurations were also tested on the RNN to find the model structure that would perform the best (Figure 7). Most of the parameters analyzed were the same as for the feedforward network, but the activation function was also analyzed here [29,30,31].

It can be observed that almost all networks have a similar MSE for the validation dataset. The model that performed the best with a validation mean squared error of 0.001006 has 10 hidden units and a sigmoid activation function. The dense layer has 8 units and a hyperbolic tangent activation function. Out of the three optimizers, Adam was again the one that performed the best, using a learning rate of 0.06896. For the learning reduction algorithm, the monitor value was val_mse, and the value 15 was the best for the patience parameter. The network was trained for 150 epochs with a batch size of 8. 

## 4. Results

All the models were tested on the same dataset containing 315 analytically generated samples with damage cases, to be able to objectively compare them. A comparison can be seen in Table 3. As we can see from the findings for the basic network, it was noted from the start that having one model train on the whole beam length would not always be very effective. Splitting the data for the beam into 9, 10, or 14 segments and then training the models and stacking them proved to yield better results, with the model with 14 networks yielding the best results. In this configuration, three models were trained on the same beam section and then stacked to generate the final prediction. A total of 2 of the 14 models, 1 for the fixed end and 1 for the free end of the beam, were introduced explicitly because it was observed that the networks have trouble creating accurate predictions for these areas. Accuracy was improved for the extremities, but it remained the section where the networks had the highest prediction errors. Table 4 displays a comparison of the various network types, overlapping techniques, and results.

The basic model performed poorly, having significant errors, and for 40 samples, the location was inaccurately predicted by more than 10 mm, which is deemed a substantial error, especially considering the objective of automatically detecting defects without requiring further inspection. We observed big accuracy improvements after overlapping the trained segments and creating nine models. For this overlapping scenario, the BiLSTM performed very well, with only three samples having an error above 1%. 

The predictions continued to improve when adding more overlap and ten trained models. Another interesting fact to note is that the ANN model starts to perform better than the RNN models. This could be attributed to the forget gate structure, where the gate controls which new information should be learned. Hence, by increasing the amount of training data, the model becomes capable of learning and generating more accurate predictions. When training 14 models and employing the stacking technique, all the models perform very well without any error above 1%. 

**Figure 8:**
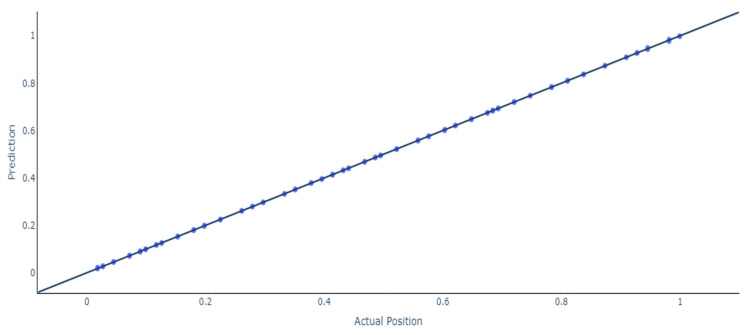
Actual vs predicted location using feedforward ANN.

Additionally, the same trend can be observed for this scenario because the ANN outperforms the RNN and LSTMs models, having a test mean squared error of 0.0000006 with more than 273 samples being approximated under 1 mm and only 6 having a deviation of more than 2 mm. Furthermore, an in-depth analysis of the results for the models with 14 stacked feedforward ANNs and Bidirectional LSTMs is presented. Figure 8 shows the results and the deviations between the actual and the predicted location of the stacked model using feedforward ANN. 

**Figure 9:**
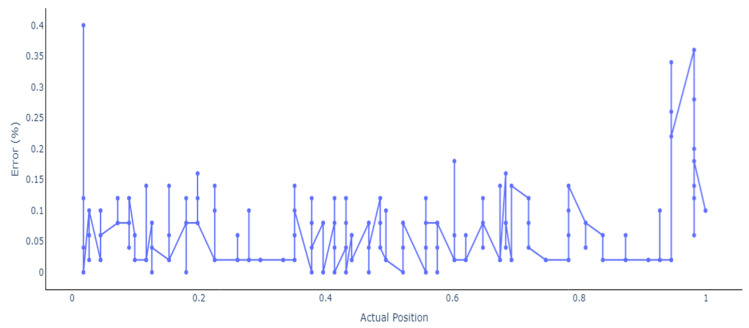
Error percentage in model prediction using feedforward ANN.

The error percentage for the test dataset is shown in Figure 9, where it can be observed that the locations are not predicted with sufficient accuracy at the start and at the end of the beam. The biggest error is 0.4% for location 0.0018, followed by 0.36% and 0.28% for 0.981, 0.34%, and 0.26% for 0.945, as shown in Table 4.

Compared to the basic neural model, whose most significant error was 25.74%, the tuned stacked model significantly improved performance. Additionally, apart from 11 samples, the error was less than 0.2% for all the other cases, and for 267 samples, the error was less than 0.1%, indicating that the model can accurately predict the location of the defect within 1 mm.

**Figure 10:**
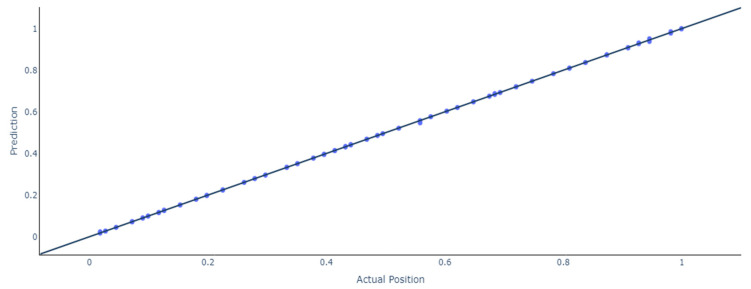
Actual vs. predicted location using Bidirectional LSTM.

Furthermore, the results for the bidirectional LSTM are shown in Figure 10. Apart from some damage locations that have been estimated with an error greater than 0.2%, the rest of the locations are accurately predicted. Like the feedforward model, the locations at the beginning and at the end of the beam seem to be harder to predict and where most of the errors are. Regardless of the number of layers, neurons, or for how many epochs the network was trained, the same behavior could be seen in all trained models, where the locations at the end have the highest error percentage.

**Figure 11:**
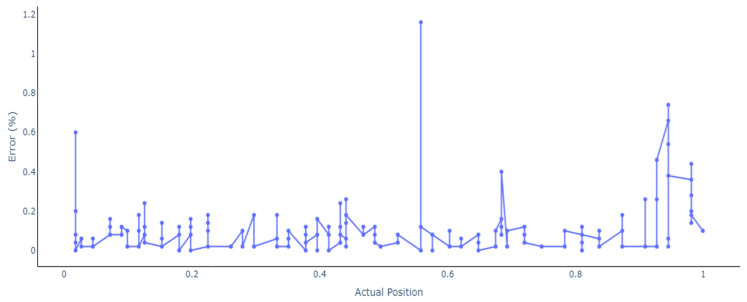
Error percentage in model prediction using Bidirectional LSTM.

Figure 11 shows the error percentages and gives an accurate view of where the model cannot predict the locations accurately. The greatest error of 0.6% is for point 0.018, and the other locations with high errors are at the end of the beam with 0.58% and 0.54% for 0.945. Other than for the locations near the end of the beam, only for point 0.684 is there an error higher than 0.3%. Table 5 summarizes these results.

The bidirectional LSTM model performed similarly to the Multilayer Perceptron, with 14 samples having an error higher than 0.2% and 251 samples with an error less than 0.1%. Compared to the error of 0.0000006 achieved with the feedforward ANN, the overall mean squared error for Bidirectional LSTM was a little higher at 0.0000012.

## 5. Results for FEM and Measured Data

Since the stacked model containing 14 feedforward ANNs performed the best on generated data, we only consider that model in this section. In addition to the generated test dataset, containing 315 samples, we gathered a test dataset using FEM, which contains 10 samples presented in Table 6. Additionally, experimental measurements were conducted using an experimental setup [15]. The eight RFS values based on different vibration modes are used as inputs to predict the defect position. Table 7 shows the predicted location together with the error. The model had only two samples with an error slightly over 1% as the other samples had an error of around 0.5%, with the lowest being 0.2% and 0.18% having most of the absolute errors around 5 mm and less.

Furthermore, the data from experimental measurements made on four beams, presented in Table 8, were tested and analyzed to obtain a better understanding of the model’s performance and to find out which aspects might need some improvement in the future. Table 9 presents the results using the ANN models. The highest error percentage of 1.9% was for position 0.395, which is still far less than similar studies have achieved [32,33].

In summary, all the network types have proven to be able to predict the damage location, with the stacked model containing 14 feedforward ANNs being the model that performed the best, with more than 86% of the predicted locations being within 1 mm of the actual damage location. This model also proved to have the best performance on the test datasets generated using the FEM method or an experimental setup to measure the natural frequencies.

## 6. Conclusions

In this study, we investigated the problem of identifying the damage location using different neural network architectures. By leveraging the power of deep learning techniques, our model has been proven to solve the problem of damage localization accurately and efficiently.

Experimental results show that BiLSTM, which processes input both forward and backward, performed better when fewer models were stacked, as it was able to extract more comprehensive characteristics from the data. However, as the number of models increased, MLP outperformed LSTM, and BiLSTM, in deeper architectures. Specifically, when 14 models were stacked, MLP achieved the best performance, due to its ability to handle complex, multi-layer feature representations more effectively than recurrent-based models. 

As depth grows, the network can recognize more intricate damage patterns because each layer in the proposed stacked ANN model collects progressively higher-level and more abstract characteristics from the one before it. Early layers may concentrate on identifying fundamental structural abnormalities, whereas deeper layers integrate these characteristics to identify precise damage locations.

We demonstrated that the damage position can be accurately predicted through extensive experimentation and evaluation of different architectures and optimization techniques. Out of 315 samples, we achieved precise prediction within a 2 mm range for 309 samples. The trained neural network models exhibited exceptional performance, outperforming traditional methods and similar approaches that have previously been implemented.

A pre-established technique was applied to forecast the natural frequencies of the crack-affected beam to generate the training datasets. Based on the natural frequencies of the beam in a damaged and undamaged state, the RFS values for eight modes of vibrations were calculated, which were then used as input for the neural network models to predict the defect position. FEM simulations in ANSYS are performed to generate the test dataset. Additionally, an alternative experimental setup, which utilizes an accelerometer and the LabVIEW software, was employed to measure the natural frequencies of a steel cantilever beam. Subsequently, the RFS was calculated based on these measurements.

Using a stacking technique proved to bring huge accuracy improvements and allowed the creation and training of more granular models that were responsible for predicting the positions on a specific segment of the beam. The best-performing model was made up of 14 models that were trained for various segments, resulting in significantly improved accuracy in its predictions. Along with the stacking strategy, hyperparameter tuning also aids in improving model performance, as the structure of the Artificial Neural Networks strongly influences how well a model performs. Our results show that the network type that performed the best was a feedforward ANN having 2 hidden layers, one with 8 and 36 neurons, respectively. The optimizer used was Adam, who had a learning rate of 0.0427. The absolute errors in crack location fell within the range of ±1 mm for more than 86% of the test scenarios and ±2 mm for more than 98%. BiLSTM had also a similar performance of 79% samples having the absolute error in the range of ±1 mm and 95% in the range of ±2 mm. Such precision was not achieved by the current methods.

Additionally, considering that the Multilayer Perceptron performed the best, it was also evaluated on the two test datasets that were generated using FEM and an experimental setup for measurements, respectively. While the results are promising, there are still opportunities for further improvement. As neural networks continue to evolve, incorporating additional architectural enhancements and exploring advanced training strategies could enhance performance even further. 

One remark made when evaluating the model’s performance was that all the models had trouble predicting locations close to the ends of the beam. Moreover, most of the inaccurate predictions are near the right end of the beam, which is the free end. One of the reasons might be that the RFSs are close to 0 for this part of the beam, and therefore, the models could not have such an accurate prediction as for the other beam sections. Moreover, having more measurements and training data for this area could help improve the overall performance. We could expect more reliable and accurate predictions by directing our attention to these specific areas and improving the model’s understanding of the unique characteristics of the beam’s free end. As a consequence, the efficacy of damage localization systems is enhanced. Therefore, future research will be devoted to exploring new methodologies aimed at enhancing the performance of the models, as well as creating different strategies to obtain more precise measurements to improve model training.

## Figures and Tables

**Table 1:** Examples of training data.

Output Data		Input Data							
Position	Severity	Mod 1	Mod 2	Mod 3	Mod 4	Mod 5	Mod 6	Mod 7	Mod 8
0	0.000866543	0.000867	0.000867	0.000867	0.000867	0.000867	0.000867	0.000867	0.000867
0.002	0.000866543	0.000862	0.00085	0.00084	0.000829	0.000818	0.000808	0.000797	0.000787
0.004	0.000866543	0.000857	0.000834	0.000813	0.000792	0.000771	0.000751	0.000731	0.000711
0.006	0.000866543	0.000852	0.000818	0.000787	0.000756	0.000726	0.000696	0.000667	0.000639
0.008	0.000866543	0.000848	0.000802	0.000761	0.000721	0.000682	0.000644	0.000607	0.000571
0.01	0.000866543	0.000843	0.000786	0.000736	0.000686	0.000639	0.000593	0.000549	0.000506
0.012	0.000866543	0.000838	0.00077	0.000711	0.000653	0.000598	0.000545	0.000494	0.000446

**Table 2:** Comparison of actual and predicted location using baseline ANN.

Position	Predicted Position	Prediction Error (%)
0.333	0.5904	25.74
0.333	0.5328	19.98
0.027	0.1116	8.46
0.837	0.7556	8.14
0.027	0.0932	6.62

**Table 3:** Model evaluation comparison.

	Model	MSE	No. of Samples Cases	No. of Samples Cases with Error		
				>(10 mm)	>(2 mm)	<(1 mm)
	Basic ANN	0.0005013	315	40	127	129
9 Models	ANN	0.00002316	315	15	74	171
	RNN	0.00001881	315	6	77	164
	LSTM	0.00002075	315	4	82	158
	BiLSTM	0.00001341	315	3	67	177
10 Models	ANN	0.000003892	315	1	49	193
	RNN	0.000009676	315	6	69	172
	LSTM	0.000005256	315	2	40	231
	BiLSTM	0.000004014	315	2	40	231
14 Models	ANN	0.0000006	315	0	6	273
	RNN	0.000003325	315	1	33	233
	LSTM	0.000001715	315	0	17	250
	BiLSTM	0.000001277	315	0	14	250

**Table 4:** Comparison of actual and predicted location using feedforward ANN stacking.

Generated Position (m)	Predicted Position (m)	Prediction Error (mm)	Prediction Error (%)
0.018	0.022	4	0.4
0.981	0.9774	3.6	0.36
0.945	0.9484	3.4	0.34
0.981	0.9838	2.8	0.28
0.945	0.9476	2.6	0.26

**Table 5:** Comparison of actual and predicted location using Bidirectional LSTM stacking.

Generated Position (m)	Predicted Position (m)	Prediction Error (mm)	Prediction Error (%)
0.018	0.024	6	0.6
0.945	0.9508	5.8	0.58
0.945	0.9504	5.4	0.54
0.927	0.9316	4.6	0.46
0.981	0.9854	4.4	0.44

**Table 6:** FEM test dataset.

Position	Severity	Relative Frequency Shifts							
		Mod1	Mod 2	Mod 3	Mod 4	Mod 5	Mod 6	Mod 7	Mod 8
0.056	1	0.00253	0.00160	0.00094	0.00045	0.00014	−0.00001	−0.00001	0.00011
0.083	0.5	0.00157	0.00073	0.00024	−0.00003	−0.00010	−0.00003	0.00014	0.00031
0.125	1.6	0.00924	0.00223	0.00001	0.00078	0.00305	0.00495	0.00017	0.00001
0.233	1	0.00137	−0.00001	0.00079	0.00124	0.00046	−0.00003	0.00075	0.00144
0.363	0.4	0.00016	0.00016	0.00022	0.00003	0.00026	0.00016	0.00000	0.00037
0.43	0.5	0.00032	0.00056	0.00015	0.00036	0.00053	0.00005	0.00072	−0.00001
0.59	1	0.00014	0.00141	0.00066	0.00042	0.00132	−0.00003	0.00136	0.00037
0.604	0.4	0.00004	0.00027	0.00019	0.00007	0.00028	0.00004	0.00040	0.00012
0.806	1.6	0.00005	0.00081	0.00331	0.00532	0.00440	0.00151	0.00010	0.00234
0.906	1	−0.00004	−0.00001	0.00010	0.00035	0.00072	0.00115	0.00150	0.00167

**Table 7:** Results for FEM dataset using 14 stacked feedforward ANNs.

Generated Position [m]	Predicted Position [m]	Error [mm]	Error [%]
0.056	0.0508	5.2	0.52
0.083	0.0772	5.8	0.58
0.125	0.1148	10.2	1.02
0.233	0.2348	1.8	0.18
0.363	0.3672	4.2	0.42
0.43	0.4252	4.8	0.48
0.59	0.5952	5.2	0.52
0.604	0.592	12	1.2
0.806	0.8028	3.2	0.32
0.906	0.908	2	0.2
0.43	0.4228	7.2	0.72
0.59	0.5888	1.2	0.12
0.604	0.5948	9.2	0.92
0.806	0.804	2	0.2
0.906	0.9072	1.2	0.12

**Table 8:** Measurements test dataset.

Position	Severity	Mod1	Mod 2	Mod 3	Mod 4	Mod 5	Mod 6	Mod 7	Mod 8
0.31	0.8	0.0012	0.0007	0.0018	0.0005	0.0004	0.0019	0.000487	5.81 × 10^−5^
0.587	1.2	0.0002	0.0044	0.002	0.0015	0.0039	0.0022	0.001589	0.000388
0.395	1.2	0.002	0.0028	0.0026	0.0006	0.0046	0.0001	0.001149	0.00107
0.795	2	0	0.002	0.0103	0.0135	0.0071	0.0006	8.51 × 10^−5^	0.000978

**Table 9:** Results for experimental measurements dataset using 14 stacked feedforward ANNs.

Generated Position	Predicted Position	Error [%]
0.31	0.3152	0.52
0.587	0.596	0.9
0.395	0.376	1.9
0.795	0.7832	1.18

## Data Availability

Data is contained within the article. The original contributions presented in the study are included in the article, further inquiries can be directed to the corresponding author.
